# Supplementary material for: Dynamics of venom composition across a complex life cycle
Source: eLife. 2018 Feb 9;7:e35014. doi: 10.7554/eLife.35014 (PMC5832418; doi:10.7554/eLife.35014)
Supplement: Transparent reporting form [file elife-35014-transrepform.docx]

***eLife’s* transparent reporting form**

We encourage authors to provide detailed information *within their submission* to facilitate the interpretation and replication of experiments. If you have any questions, please contact us: [editorial@elifesciences.org](mailto:editorial@elifesciences.org).

**Sample-size estimation**

- You should state whether an appropriate sample size was computed when the study was being designed
- You should state the statistical method of sample size computation and any required assumptions
- If no explicit power analysis was used, you should describe how you decided what sample (replicate) size (number) to use

Please outline where this information can be found within the submission (e.g., page numbers or figure legends), or explain why this information doesn’t apply to your submission:

For the sea anemones, because the material was abundant and the sea anemones are grown in the lab in very large numbers, each sample was prepared from five or more adults or hundreds of larvae in order to normalize for any individual variation. This is described in page 14, lines 33-34 and page 15 lines 19-20. Zebrafish larvae were used in toxicity tests in 3 groups of 5-7 fish per treatment as described in page 20, lines 5-8. As for the shrimp, they were collected in high abundance at each location. Preliminary screening of control group injections was used to determine survivability of bovine serum albumin (BSA), resulting in 100% survivability. Without a negative impact of shrimp injections we decided to do 10 individuals/treatment to ensure we would have sufficient numbers to observe a response following injection. These numbers were comparable to what had been published previously (Nedosyko et al. 2014, PLoS ONE). The shrimp injections are described in page 20, lines 10-12. For *Fundulus* larvae, interactions were done with several stages of sea anemones: eggs, egg packages, planula larvae, and primary polyps. These were done in replicates of 3, with 5 fish in each replicate. Based on these interaction experiments, we determined that the primary polyps may be impacting their vertical position, but we were unable to quantify this using 24 well plates. To further evaluate these interactions an aquarium was constructed to visualize fish larvae’s vertical position relative to primary polyps. Preliminary results indicated that there was a high rate of individual variation across fish larvae and we decided to test 10 fish for each treatment. The fish larvae and primary polyp interactions are described in page 11, line 32 – page 12, line 8, and page 21 lines 3-17. Interactions between different sea anemone life stages and adult *Fundulus* were anecdotal (not statistical) with three fish for each developmental stage tested. The description of how adults were handled is on page 19, lines 27-34.

**Replicates**

- You should report how often each experiment was performed
- You should include a definition of biological versus technical replication
- The data obtained should be provided and sufficient information should be provided to indicate the number of independent biological and/or technical replicates
- If you encountered any outliers, you should describe how these were handled
- Criteria for exclusion/inclusion of data should be clearly stated
- High-throughput sequence data should be uploaded before submission, with a private link for reviewers provided (these are available from both GEO and ArrayExpress)

Please outline where this information can be found within the submission (e.g., page numbers or figure legends), or explain why this information doesn’t apply to your submission:

The number of biological and technical replicates can be found in pages 14-16, and 20-21 (materials and methods).

**Statistical reporting**

- Statistical analysis methods should be described and justified
- Raw data should be presented in figures whenever informative to do so (typically when N per group is less than 10)
- For each experiment, you should identify the statistical tests used, exact values of N, definitions of center, methods of multiple test correction, and dispersion and precision measures (e.g., mean, median, SD, SEM, confidence intervals; and, for the major substantive results, a measure of effect size (e.g., Pearson's r, Cohen's d)
- Report exact p-values wherever possible alongside the summary statistics and 95% confidence intervals. These should be reported for all key questions and not only when the p-value is less than 0.05.

This information is found in the legend of Figure 1 (pages 25-26) and in the materials and methods (pages 15, 21)

Please outline where this information can be found within the submission (e.g., page numbers or figure legends), or explain why this information doesn’t apply to your submission:

(For large datasets, or papers with a very large number of statistical tests, you may upload a single table file with tests, Ns, etc., with reference to page numbers in the manuscript.)

**Additional data files (“source data”)**

- We encourage you to upload relevant additional data files, such as numerical data that are represented as a graph in a figure, or as a summary table
- Where provided, these should be in the most useful format, and they can be uploaded as “Source data” files linked to a main figure or table
- Include model definition files including the full list of parameters used
- Include code used for data analysis (e.g., R, MatLab)
- Avoid stating that data files are “available upon request”

Please indicate the figures or tables for which source data files have been provided:

Source data are available as an Excel sheets (Supplementary tables S1 and S2) for the data presented in Figure 1.
